# Corneae from body donors in anatomy department: valuable use for clinical transplantation and experimental research

**DOI:** 10.1186/s12886-020-01546-2

**Published:** 2020-07-13

**Authors:** Cristina Martin, Thomas Tschernig, Hamon Loic, Loay Daas, Berthold Seitz

**Affiliations:** 1 Department of Ophthalmology, Saarland University Medical Center (UKS), Homburg, Germany; 2 Institute of Anatomy and Cell Biology, Campus Homburg/Saar, Saarland University, Kirrberger Street, Building 61, 66424 Homburg/Saar, Germany

**Keywords:** Cornea transplantation, Body donors, Donor cornea, Anatomy, Eye bank

## Abstract

**Background:**

Explanted corneae are highly needed for the surgical management of patients with severe corneal diseases. The aim of this study was to determine whether the body donors from the Institute of Anatomy are a suitable source of donor corneae.

**Methods:**

At the Institute of Anatomy at Saarland University Medical Center in Homburg, corneae are prelevated from body donors who had consented to the removal of tissues for transplantation purposes during their lifetime. Following the report of death, the LIONS Eye Bank is informed and the contraindications of corneal explantation are clarified. Obtaining a blood sample within 24 h postmortem is mandatory.

**Results:**

The Institute of Anatomy had 150 body donors in the time period from January 2018 to June 2019. Out of these, 68 (45.3%) were reported to the Eye Bank. The age of the donors (median 82 years (range: 57–96)) is not critical since the quality of the corneae depends on the number of endothelial cells (mean: 2109 ± 67 cells/mm^2^ (range: 511–2944 cells/mm^2^)). Contraindications were present in 19 (12.6%) cases. The corneae were extracted from 49 (32.7%) body donors. Out of these 98 corneae, 46 (46.9%) were successfully transplanted. Of all non-transplanted corneae, 6 (6.1%) were microbiologically contaminated, 10 (10.2%) had a positive serology, 22 (22.5%) had an endothelial cell count < 2000 cells/mm^2^ and 6 (6.1%) are at time of this analysis still in culture medium. The non-transplanted tissues were used for research.

**Conclusions:**

Explanted corneae from the Institute of Anatomy are a valuable option in obtaining grafts for corneal transplantation, which is why we are working toward on expanding cooperation with this department.

## Background

Blindness due to corneal diseases represents a major public health burden that is estimated, according to the World Health Organization, to affect 1.5 million people worldwide [1]. With the first successful corneal transplantation performed more than a century ago, a new era began in the treatment of various corneal diseases. Keratoplasties have been performed successfully since then and are today the most frequent type of transplantation performed in human beings [2, 3].

**Fig. 1:**
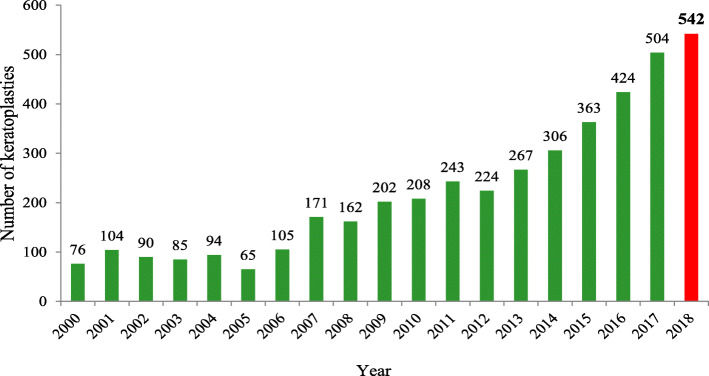
The development of keratoplasties at the Department of Ophthalmology, Saarland University Medical Center (UKS), Germany. Number of keratoplasties at the Department of Ophthalmology, Saarland University Medical Center (UKS), Germany, since the foundation of the LIONS Eye Bank Saar - Lor - Lux, Trier/Westpfalz

This success translated immediately in an ongoing increase in corneal transplantation worldwide. In the United States of America alone, techniques like lamellar keratoplasty showed an astonishing increase from 5 to 58% of all corneal transplantation in the past decade [4]. In Germany, the number of reported keratoplasties increased from 4730 in 2001 to 7325 in 2016 by a factor of 1.5, mainly due to an increase in the number of posterior lamellar keratoplasties [5]. An increased number of performed keratoplasties from 76 in 2000 to 542 in 2018 was also observed at our Department of Ophthalmology, Saarland University Medical Center (SUMC) in Homburg (Fig. 1).

Despite all the remarkable progress that characterises corneal transplantation, there continues to be a tremendous number of patients worldwide who are in need of a donor cornea. The Global Survey of Corneal Transplantation and Eye Banking showed that worldwide about 53% of the population has no access to corneal transplantation, with only 35.7% having satisfactory access to donor corneae [6]. Generally speaking, the worldwide corneal supply is very scarce with only 1 available cornea for every 70 needed [6]. This shortage of donor corneae affects Germany as well, where it is estimated that around 4000 patients are annually on the waiting list for a corneal transplant [5].

There are many identified factors that may contribute to the present corneal graft scarcity including: lack of efficient notification system of potential donors [7], reduced number of eye banks with only 16 countries worldwide having more than 5 eye banks [6], lack of information and misconceptions [1], culture and religion [1]. One way to manage the problem of corneal graft scarcity is to identify new sources of corneae. Such sources may include human corneae as well as corneal stromal substitutes including synthetic inert prostheses, acellular scaffolds with and without enhancement of endogenous regeneration and cell-based replacements [8].

**Fig. 2:**
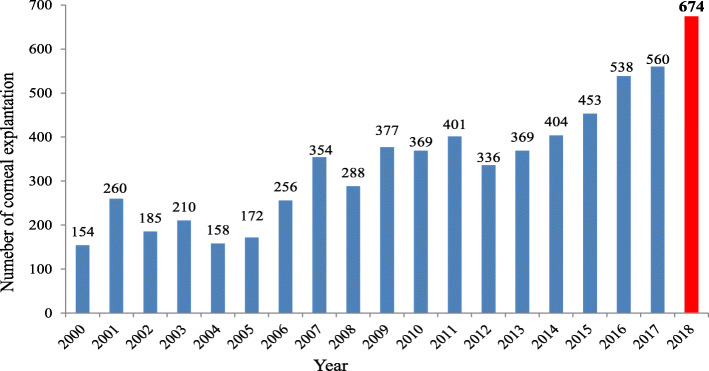
The development of corneal explantations at the Department of Ophthalmology, Saarland University Medical Center (UKS), Germany. Number of corneal explantations at the Department of Ophthalmology, Saarland University Medical Center (UKS), Germany, since the foundation of the LIONS Eye Bank Saar - Lor - Lux, Trier/Westpfalz

The local demand on donor corneae in our Department of Ophthalmology at SUMC in Homburg is very high. The waiting list of 350 patients remains stable for years despite an increased number of corneal excisions performed by the LIONS Eye Bank (Fig. 2). In order to keep up with the increasing need of donor corneae, the Department of Ophthalmology at the SUMC in Homburg continuously improves its collaboration with other ophthalmological centers in Germany, foreign countries such as Luxemburg and with the Institute of Anatomy at the University of Saarland. Such a collaboration between an ophthalmological department′s Eye Bank and an Institute of Anatomy is not routinely seen in Germany. To our knowledge there are very few centers in Germany that carry such a collaboration and to date no data on the results of the collaboration have been published.

Therefore, the aim of this study focuses on determining the role of the Institute of Anatomy as an alternative source of donor corneae as well as the evaluation of the suitability of obtained corneal grafts for transplantation.

## Methods

### Study design and participants

Data for this study was collected retrospectively from January 2018 to June 2019 from body donors from the Institute of Anatomy who consented to the prelevation of tissues for transplantation purposes during their lifetime. Contraindications for prelevation included among others: positive serology for HIV, syphilis, hepatitis, multiresistent bacterial infections, hematological neoplasia etc. An extended list of contraindications to cornea prelevation is provided in Table 1. In the absence of contraindications, a physician from the transplantation team performed the corneal excision following the local and international standards for organ removal. Cases reported later than 24 h after declared death were not accepted as potential corneal donors and, therefore, not included in this study. All data were collected anonymously and included donor age, sex, endothelial cells number, contraindications to corneal excision, presence of infections in the culture media and serology for HIV and hepatitis viruses. The whole procedure was in accordance with the regulations of the section “Tissue Transplantation and Biotechnology” of the German Ophthalmological Society.

**Table 1:** Extended list of contraindications to cornea prelevation at the Department of Ophthalmology, Saarland University Medical Center (UKS), Germany

*Number*	*Contraindications*
*1.*	Unknown cause of death or significant disease of unknown etiology in the medical history
*2.*	Viral donor diseases: Infection with HIV, hepatitis B/C, HTLV I/II
*3.*	Bacterial donor diseases: Syphilis or other chronic persistent bacterial infections (brucellosis, typhus, rickettsioses, leprosy, relapsing fever, tularemia)
*4.*	Protozoonotic donor diseases: Babesiosis, trypanosomiasis (e.g., Chagas disease), Leishmaniasis
*5.*	Active systemic infections: bacterial, viral, fungal, parasitic or of unclear etiology
*6.*	Fungal sepsis or sepsis with multi-resistant germs (a bacterial sepsis with usual spectrum is not a contraindication)
*7.*	Central nervous disorders of unknown cause: (M. Alzheimer, M. Parkinson, unclear fast progressive Dementia, Multiple Sclerosis, Amyotrophic Lateral Sclerosis)
*8.*	Hematological neoplasias, leukemias, lymphomas
*9.*	Ophthalmic donor diseases with visible change in the cornea (corneal surgery, local infections, tumors of the eye)
*10.*	Risk of disease transmission by prions: recipients of dura mater, cornea, sclera, hetero- or xenografts; recipients of pituitary hormones
*11.*	Premortal uptake of substances that by transplanting can lead to a harmful effect (poisons, heavy metals)
*12.*	Donors who had premortal blood transfusion in the last 48 h with a limit of 22.5 ml per kg of body weight
*13.*	Time-limited exclusion: 2 years after healing: Salmonellosis, Q fever; Tuberculosis, Leptospirosis; 4 years after the cure of Malaria; 4 weeks after healing of Measles, Rubella, VZV
*14.*	Risk for ZIKA virus infection: (clinical signs of infection or stay within the last 28 days before death in a Zika virus endemic area (Uganda, Africa, Asia Micronesia, French Polynesia, Brazil, Columbia, Venezuela, Central America))
*15.*	Infections with MRSA / ESBL / VRSA

### Statistical analysis

The collected data included numerical and categorical variables. Numerical variables are reported as mean and standard error of the mean. Categorical data is presented as absolute number and percentage. Comparison of the mean was performed using t-test for numerical data and chi-square test for categorical data. Correlation analysis was performed using Pearson correlation. All the hypotheses were tested at a level of significance of 0.05. The Bonferroni method was applied to adjust for multiple comparison. The statistical analyses were performed using SPSS (IBM, version 24).

## Results

From January 2018 to June 2019, the Institute of Anatomy in Homburg/Saar accepted 150 (100%) body donors. During this period, 68 (45.3%) donors, of which 43 (28.7%) in 2018 and 25 (16.6%) in the first 6 months of 2019, were reported to our Eye Bank. The remaining 82 (54.7%) cases were excluded from this study as they were announced to the Eye bank after 24 h postmortem or they did not meet the donor inclusion criteria (Table 1).

**Fig. 3:**
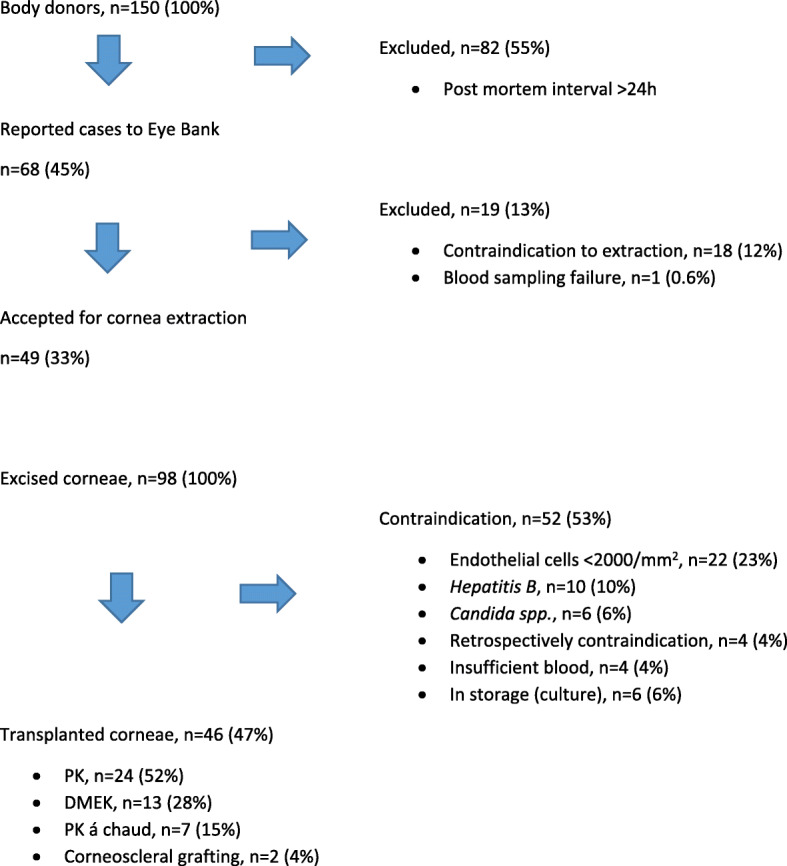
Schematic presentation of study results (January 2018 – June 2019)

Out of the 68 cases that were announced as potential donors, the Eye Bank explanted corneae from 30 (44.2%) body donors in 2018 and 19 (27.9%) in 2019. The remaining 19 (27.9%) body donors either presented contraindications (Table 1) or family members denied the excision of corneae (Fig. 3).

**Fig. 4:**
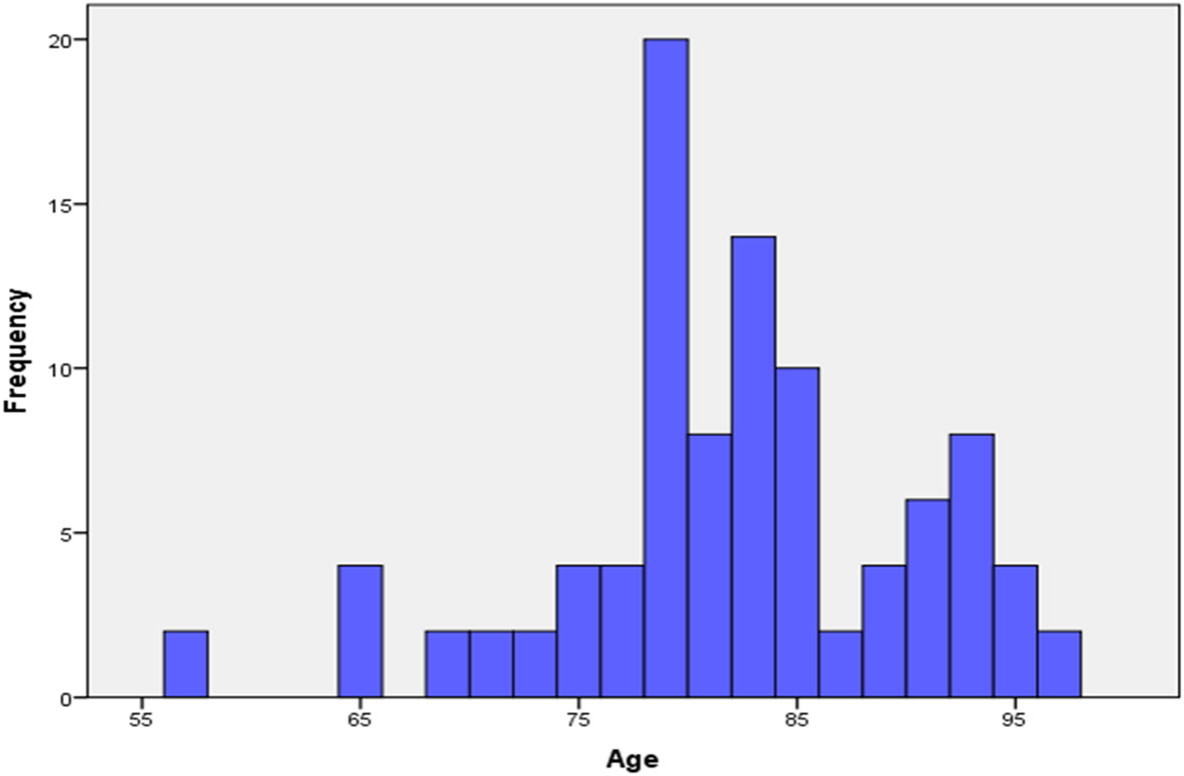
Age distribution of body donors. Distribution of age in 49 included body donors from the Institute of Anatomy

Median age of the 49 included body donors was 82 years (range 57–96 years). Figure 4 represents the distribution of age in body donors. The body donors’ female to male ratio was 1.4: 1, with females representing 59.1% of body donors. Regarding cause of death, 10 (20.3%) body donors died of a cardiogenic shock and 8 (16.4%) of multiorgan insufficiency. Distribution of cause of death is represented in Table 2.

**Table 2:** Distribution of death cause of all 49 body donors from the Institute of Anatomy

Death cause in body donors	Frequency n. (%)
Cardiogenic Shock	10 (20.3%)
Multiorgan insufficiency	8 (16.4%)
Heart insufficiency	7 (14.4%)
Pneumonia	4 (8.3%)
Lung Cancer	3 (6.2%)
Respiratory insufficiency	2 (4.1%)
Stroke	2 (4.1%)
Sepsis	2 (4.1%)
Not defined	2 (4.1%)
Liver metastasis	1 (2.0%)
Lung oedema	1 (2.0%)
Malnutrition	1 (2.0%)
Atrial fibrilation	1 (2.0%)
Lung embolie	1 (2.0%)
Encephalopathie	1 (2.0%)
COPD	1 (2.0%)
Cholangitis	1 (2.0%)
Urosepsis	1 (2.0%)
Total	49 (100%)

Of all 98 prelevated corneae, 46 (46.9%) could be used for transplantation, 22 (22.4%) had endothelial cell damage, 10 (10.2%) showed a positive serology for hepatitis B virus, 6 (6.1%) were microbiologically contaminated with *Candida albicans* in the culture and in 4 (4.1%) corneae, contraindications to excision were found retrospectively. 4 (4.1%) corneae could not be used because of insufficient blood samples needed for serology testing. At the time of this analysis, 6 (6.1%) corneae were still in culture medium (Fig. 3).

Compared to cornea from other sources, corneae from the Institute of Anatomy had statistically significant higher rates of endothelial cell damage (22.4% vs. 12.5%, *p* < 0.01) and positive serology for hepatitis B virus (10.1% vs. 3.0%, *p <* 0.01). Of the two variables, only endothelial cell damage rates showed a statistically significant Correlation with the donor age (Pearson Correlation = 0.219, *p* = 0.03). The rate of Contamination with *Candida spp.* in the culture was significantly lower in the corneae from the Institute of Anatomy (6.1% vs. 14.0%, *p* < 0.05).

All 46 (46.9%) corneae with contraindications to transplantation were used as educational or scientific material. Regarding corneae from other sources than the Institute of Anatomy, the transplantation rate at our Department of Ophthalmology reached 84.5% in 2018. The difference between the transplantation rate of corneae from the Institute of Anatomy and all other sources was statistically significant in our study (46.9% vs. 84.5%, *p* < 0.01). Corneae from the Institute of Anatomy had also a higher contraindication rate compared to other sources (46.9% vs. 15.5%, *p* < 0.01).

**Fig. 5:**
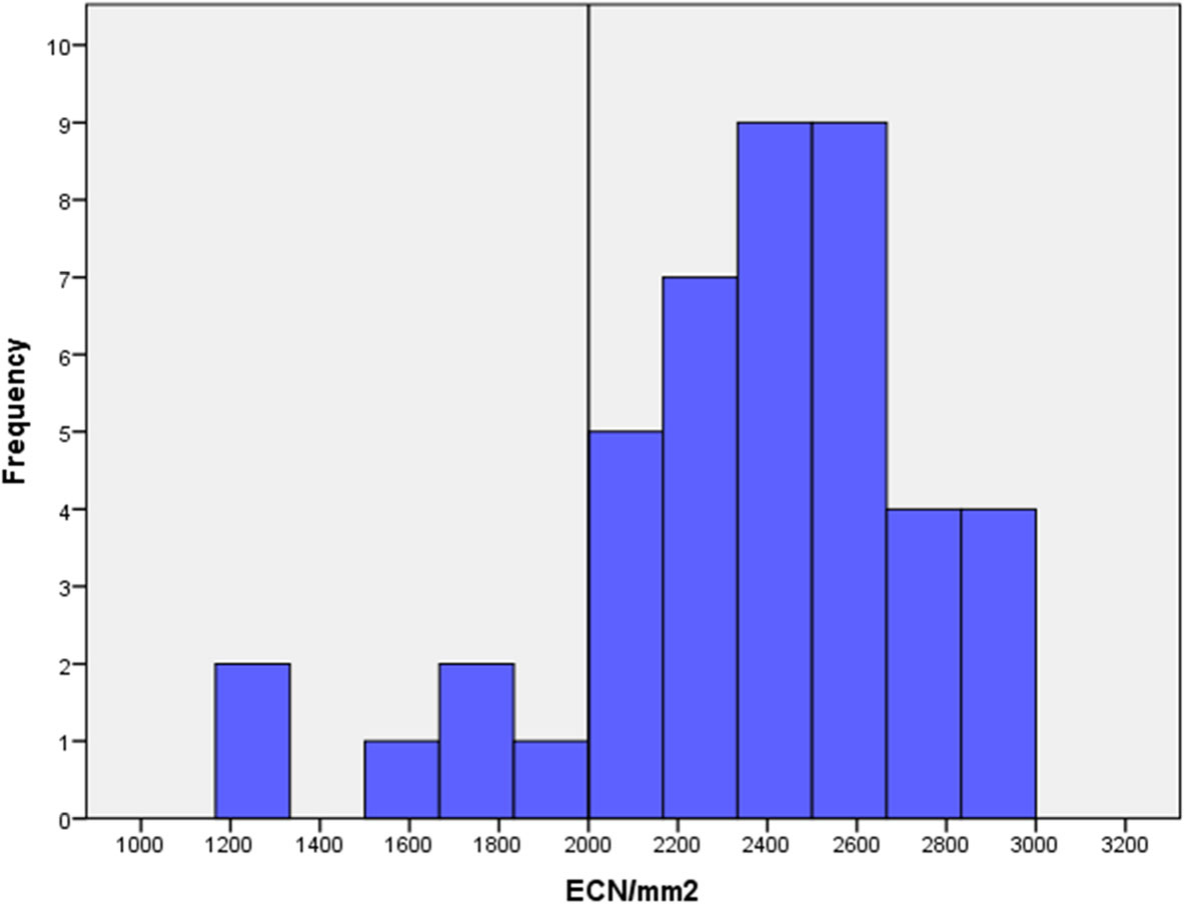
ECN in transplanted corneae. Distribution of endothelial cells number (ECN) in transplanted corneae from the body donors of the Institute of Anatomy

Following corneal excision, the endothelial cells number (ECN) was analysed. Data on ECN was present in 71 (72.4%) corneae. ECN was not analysed in 27 (27.6%) corneae with contraindications. Mean ECN was 2109 ± 67 cells/mm^2^ with a range from 511 cells/mm^2^ to 2944 cells/mm^2^. 25 (25.5%) corneae had an ECN < 2000 cells/mm^2^. In this group mean value of ECN was 1431 ± 67 cells/mm^2^, with a range from 511 cells/mm^2^ to 1995 cells/mm^2^. ECN could be obtained in 44 (95.6%) of the 46 transplanted corneae. Data on ECN in 2 (4.4%) corneae was missing. Corneae used for transplantation had a mean ECN of 2350 ± 57 cells/mm^2^. ECN in transplanted cornea ranged from 1314 cells/mm^2^ to 2944 cells/mm^2^ (Fig. 5). The ECN in transplanted cornea did not correlate with donor age (Pearson Correlation = − 0.76, *p* = 0.141).

The operative techniques, in which excised corneae were used, included: 24 penetrating keratoplasty (PKP (52.3%)), 13 Descemet Membrane Endothelial Keratoplasty (DMEK (28.2%)), 7 penetrating keratoplasty à chaud (15.2%) and 2 corneoscleral grafts (4.3%) (Fig. 3).

## Discussion

Current data suggest a permanent increase in the number of keratoplasties performed worldwide [6]. The supply of donor corneae is generally lower, with an estimated 12.7 million people waiting for a donor cornea and just 1 available cornea for 70 needed [6].

The number of performed keratoplasties as well as the number of patients on a waiting list for a corneal transplantation has increased continuously over the past decade in Germany [5]. In order to overcome this deficient supply of donor corneae, the Department of Ophthalmology in SUMC in Homburg/Saar and the LIONS Eye Bank Saar - Lor - Lux, Trier/Westpfalz established a collaboration with the Institute of Anatomy of the Medical University of Saarland in Homburg [9]. The extent of achieved collaboration is reflected in the number of excised and transplanted corneae from the Institute of Anatomy. In 2018, a total of 399 deceased patients were reported to the Lions Eye Bank in Homburg. From January 2018 to June 2019 the Institute of Anatomy contributed with 68 (17.1%) reported cases to this number. Of the 674 excised corneae at our department in 2018, 60 (8.9%) came from the Institute of Anatomy. In the same year, there were 542 performed keratoplasties at our department, of which 22 (4.1%) using corneae from the Institute of Anatomy. Although, the numerical contribution to our donor corneae pool may seem small, the implications for every corneae recipient are significant.

The transplantation rate of corneae from the Institute of Anatomy was statistically significant lower compared to corneae supplied from all other sources. Furthermore, 46.9% of corneae from the Institute of Anatomy were declared as unsuitable for transplantation purposes in our study. This represents a 3-fold increase in unsuitable corneae compared to all other sources. Compared to these results, the most recently published data by the European Eye Bank Association (EEBA) categorized 31% of the excised corneae unsuitable for transplantation purposes [10]. There are many factors that may have contributed to the observed differences. These factors are categorized as donor- and cornea-related. As for the donor specific factors, age was found to be associated with graft survival [11, 12]. This is mainly due to an initial lower ECN and greater endothelial cell loss observed with increased donor age [12]. Taking these observations into account, we hypothesized that age could have influenced the transplantation rate of the cornea from the Institute of Anatomy. In our study, the median age of cornea donors from the Institute of Anatomy was statistically significantly above the age of the donors from all other sources (82 years vs. 74 years, *p* < 0.01). Increased donor age did not correlate with the ECN in transplanted cornea (Pearson Correlation = − 0.176, *p* = 0.141). These results confirm that increased donor age was independently of ECN associated with lower transplantation rates in the corneae obtained from the Institute of Anatomy. An increase in contraindication rates along with donor age could explain the observed differences in transplantation rates. Our study showed that increased donor age is statistically significant associated with endothelial cell damage (Pearson Correlation = 0.219, *p* = 0.03). Endothelial cell damage rate could therefore be a cofounder in the donor age and transplantation rate correlation.

Donor related factors include positive donor serology. Corneae obtained from the Institute of Anatomy were more often explanted from patients infected with hepatitis B virus compared to other cornea donor sources (10.1% vs. 3.0%, *p* < 0.01). The positive serology rate in our study is consistent with the results included in the EEBA, which showed a positive serology in 9% of cases [10].

An important donor related factor with an impact on the transplantation rate is the corneal culture contamination. The culture infections with *Candida spp.* in our study showed a reduced incidence in corneae obtained from the Institute of Anatomy compared with all other sources used at our Department of Ophthalmology (6.1% vs. 14.0%, *p* < 0.05). This difference in culture contamination could be attributed to the fact that the highest contamination rate is usually found in corneae procured in clinical departments [13]. A similar rate of culture contamination was published in the literature, confirming therefore that the Institute of Anatomy, as a procurement site, did not influence the rate of corneal transplantation in our study [10, 14].

**Fig. 6:**
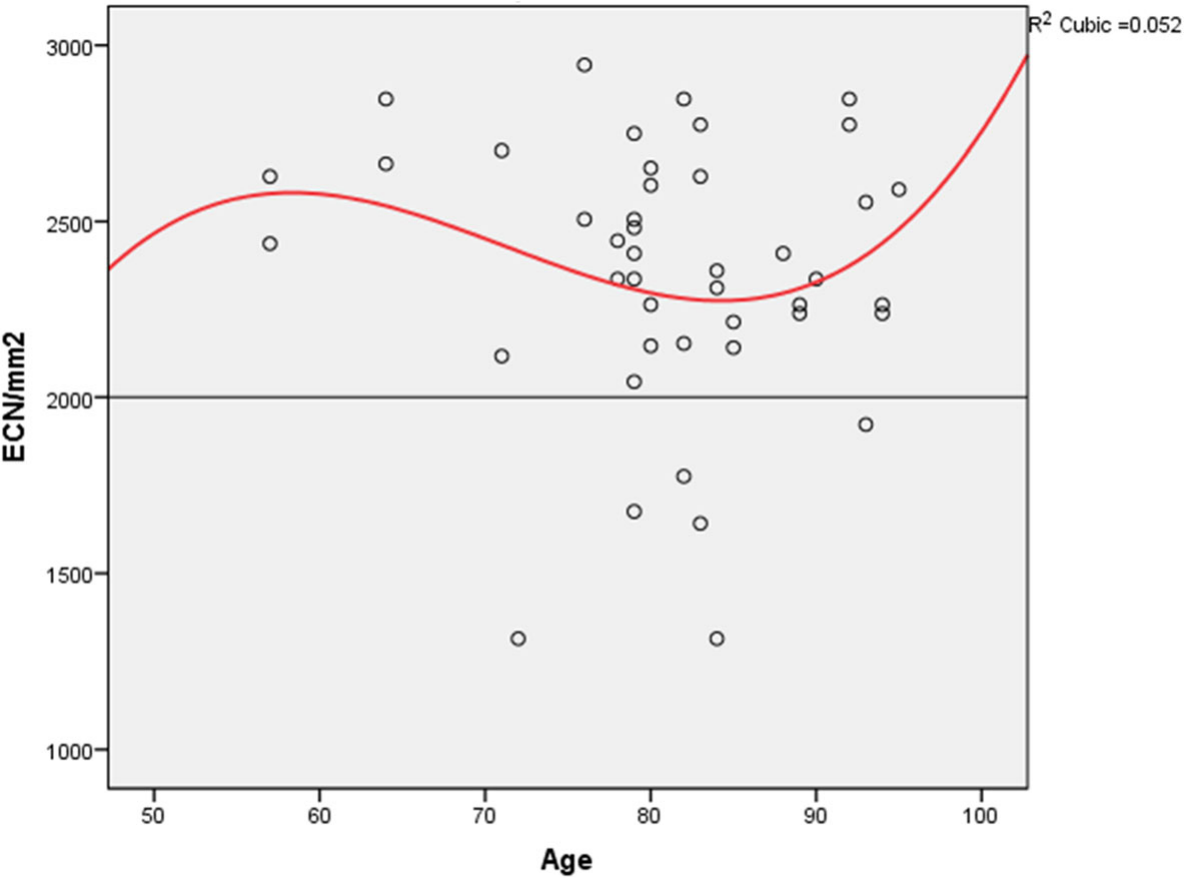
ECN and donor age correlation in the transplanted corneae. Correlation between the endothelial cell number (ECN) and the age of transplanted corneae from the body donors of the Institute of Anatomy

Cornea related factors with implications on transplantation rate include the endothelial cell number. In our study, excised corneae from the Institute of Anatomy had a lower mean ECN compared to other corneae sources (2109 ± 67 cells/mm^2^ vs. 2424 ± 17 cells/mm2, *p* < 0.05). Furthermore, our data showed similar results regarding the mean ECN in transplanted corneae (2350 ± 57 cells/mm^2^ vs. 2566 ± 15 cells/mm^2^, *p* < 0.01) (Table 3). This difference could be explained by the older age of body donors from the Institute of Anatomy, although no correlation between age and mean ECN in the transplanted cornea was observed (Fig. 6). This difference in mean ECN could translate into higher rates of functional decompensation of the grafts, since according to the linear model of endothelial cell loss, a lower ECN at the time of surgery is associated with a higher speed of endothelial cell loss over time [15]. Although, endothelial cell number of the donor was identified in many studies as a risk factor for endothelial decompensation, it is by far not the only one. Many recipient factors (age, glaucoma, smoking), surgical factors (operation time, graft size) and donor factors (age, sex) seem to play an important role in the survival of the graft [16].

**Table 3:** Differences between corneae from the Institute of Anatomy and other procurement sites

Parameter	Procurement site of corneae		***p***-value
	Institute of Anatomy	other corneae sources	
Mean ECN in all excised corneae, cells/mm^2^	2109 ± 67	2424 ± 17	***p*** **< 0.01**
Mean ECN in transplanted corneae, cells/mm^2^	2350 ± 57	2566 ± 15	***p*** **< 0.01**
Penetrating Keratoplasty, n (%)	24 (52.3%)	321 (59.2%)	*p* = 0.37
DMEK, n (%)	13 (28.2)	163 (31.1%)	*p* = 0.68
PKP á chaud, n (%)	7 (15.2%)	54 (9.9%)	*p* = 0.26

Initial low numbers of potential corneae donors could also have an impact on the transplantation rate. A substantial number of body donors were announced to the Eye Bank later than 24 h postmortem. This reduced dramatically the number of potential cornea donors. Establishing good communication channels between our ophthalmological department and the Institute of Anatomy is crucial in overcoming this problem. The implementation of a better donor notification system will help overcome the initially relatively small number of cornea donors.

Keratoplasty techniques used with donor cornea did not differ significantly between the sources. Corneae from the Institute of Anatomy were used primary for penetrating keratoplasty, DMEK and PKP à chaud (52.3% vs. 28.2% vs. 15.2%). The low rate of PKP à chaud could reflect a good quality of the corneae obtained from the Institute of Anatomy (Table 3).

Despite a low cornea donor rate, the Institute of Anatomy contributes with a small (about 4% yearly) but important number to our donor pool. As such, the main way to cover the need of corneae at our department of ophthalmology remains through further implementing and improving the existing hospital cornea retrieval programmes (HCRP).

These results, as reflected by the current study, confirm throughout the above-mentioned limitations that the Institute of Anatomy represents a valuable alternative for obtaining donor corneae in the context of an acute global need for cornea donors. This is especially true in the context of a robust anatomy department, such as the one we cooperate with.

## Conclusions

Our study supports the supplementary role of the Institute of Anatomy in increasing the corneal donor pool. Advanced age and presence of contraindications to extraction and transplantation represent major restrictions. Further improvements in the collaboration with the Institute of Anatomy including a better donor notification system will help overcome the initially relatively small number of possible cornea donors.

## Data Availability

The dataset supporting the conclusions of this article is available on request to the corresponding author.

## Abbreviations

SUMC: Saarland University Medical Center
ECN: Endothelial cells number
PKP: Penetrating keratoplasty
DMEK: Descemet Membrane Endothelial Keratoplasty
EEBA: European Eye Bank Association
HCRP: Hospital cornea retrieval programme
